# *Treponema pallidum* promoted microglia apoptosis and prevented itself from clearing by human microglia via blocking autophagic flux

**DOI:** 10.1371/journal.ppat.1011594

**Published:** 2023-08-23

**Authors:** Yun-Ting Hu, Kai-Xuan Wu, Xiao-Tong Wang, Yuan-Yi Zhao, Xiao-Yong Jiang, Dan Liu, Man-Li Tong, Li-Li Liu

**Affiliations:** 1 Center of Clinical Laboratory, Zhongshan Hospital of Xiamen University, School of Medicine, Xiamen University, Xiamen, China; 2 Department of Clinical Laboratory, Qilu Hospital (Qingdao), Cheeloo College of Medicine, Shandong University, China; 3 Department of Laboratory, Tianjin Medical University Cancer Institute and Hospital, Tianjin’s Clinical Research Center for Cancer, Key Laboratory of Cancer Prevention and Therapy, National Clinical Research Center for Cancer, Tianjin, China; 4 Department of Dermatology, Zhongshan Hospital, School of Medicine, Xiamen University, Xiamen, China; University of Illinois, UNITED STATES

## Abstract

*Treponema pallidum* (*Tp*) has a well-known ability to evade the immune system and can cause neurosyphilis by invading the central nervous system (CNS). Microglia are resident macrophages of the CNS that are essential for host defense against pathogens, this study aims to investigate the interaction between *Tp* and microglia and the potential mechanism. Here, we found that *Tp* can exert significant toxic effects on microglia in vivo in Tg (mpeg1: EGFP) transgenic zebrafish embryos. Single-cell RNA sequencing results showed that *Tp* downregulated autophagy-related genes in human HMC3 microglial cells, which is negatively associated with apoptotic gene expression. Biochemical and cell biology assays further established that *Tp* inhibits microglial autophagy by interfering with the autophagosome-lysosome fusion process. Transcription factor EB (TFEB) is a master regulator of lysosome biogenesis, *Tp* activates the mechanistic target of rapamycin complex 1 (mTORC1) signaling to inhibit the nuclear translocation of TFEB, leading to decreased lysosomal biogenesis and accumulated autophagosome. Importantly, the inhibition of autophagosome formation reversed *Tp*-induced apoptosis and promoted microglial clearance of *Tp*. Taken together, these findings show that *Tp* blocks autophagic flux by inhibiting TFEB-mediated lysosomal biosynthesis in human microglia. Autophagosome accumulation was demonstrated to be a key mechanism underlying the effects of *Tp* in promoting apoptosis and preventing itself from clearing by human microglia. This study offers novel perspectives on the potential mechanism of immune evasion employed by *Tp* within CNS. The results not only establish the pivotal role of autophagy dysregulation in the detrimental effects of *Tp* on microglial cells but also bear considerable implications for the development of therapeutic strategies against *Tp*, specifically involving mTORC1 inhibitors and autophagosome formation inhibitors, in the context of neurosyphilis patients.

## Author summary

*Treponema pallidum* (*Tp*) is the causative agent of syphilis and poses a significant threat to the central nervous system (CNS), potentially leading to neurosyphilis. Given *Tp*’s remarkable ability to circumvent host immune defenses, comprehending its immune evasion mechanisms within the CNS is of paramount importance to devise effective treatment strategies for neurosyphilis. This study aimed to explore the interplay between *Tp* and microglia, vital immune cells residing in the CNS. Both in vivo and in vitro analyses revealed considerable toxicity of *Tp* on microglia. Furthermore, *Tp* infection was found to impede lysosomal biosynthesis, leading to a diminished pool of lysosomes available for autophagosome fusion. Consequently, the autophagic flux was disrupted, resulting in the accumulation of autophagosomes. Our findings underscore the pivotal role of autophagosome accumulation as a key mechanism exploited by *Tp* to promote microglial cell apoptosis and evade clearance within the human CNS. This investigation sheds new light on the potential immune evasion strategies employed by *Tp* in the CNS context.

## Introduction

*Treponema pallidum* (*Tp*) is the causative agent of syphilis and can disseminate to the central nervous system (CNS) causing neurosyphilis [1].

*Tp* is capable of invading various parenchymal and mesenchymal cells, including macrophages, neurons, and glial cells [2–4]. Macrophages seem to be the most important components of the immune system that are involved in the elimination of *Tp* [5,6]. Microglia, which are the resident macrophages of the CNS, can quickly respond to pathogens that invade the CNS, and the proper development and activity of the CNS critically depend on microglial function [7]. Studies have shown that microglia may be involved in the pathogenesis of neurosyphilis. Mirco *et al*. found that neurosyphilis patients exhibited hypointensity in cortical susceptibility-weighted imaging, and the authors hypothesized that cortical low-density signalling in patients with neurosyphilis may be associated with abnormal microglial function [8]. In addition, the findings of Qian Yu *et al*. suggest that there is an increased number of M2a/M2c microglia in intracranial syphilitic gummas, which may be part of the mechanisms by which *Tp* escapes the host immune system [9]. However, these studies did not further explore the effect of *Tp* on microglial function, and the mechanism underlying the relationship between *Tp* and important immune defences processes in microglial cells is still unclear.

Notably, impaired autophagy has been associated with both immune senescence and chronic inflammation in macrophages. Previous studies from our laboratory showed that the membrane protein Tp47 of *Treponema pallidum* stimulates autophagy and induces cell death in human HMO6 microglial cells through the involvement of the mechanistic target of rapamycin complex 1 (mTORC1) pathway [10]. In addition, Tp47 induces autophagy to promote phagocytosis in human HMC3 microglial cells [11].

However, the membrane protein Tp47 from *Tp* cannot mimic the role of *Tp* in the disease progression of neurosyphilis. In addition, the changes in microglial function that occur in response to *Tp* stimulation and the possible impact of disordered autophagy on the function and survival of microglia are not clear. Hence, it is imperative to undertake additional investigations aiming to unravel the exact involvement of *Tp* in the intricate mechanisms pertaining to microglial autophagy and its impact on microglial cells. Such an in-depth comprehension will significantly contribute to the advancement of our understanding regarding the progression of neurosyphilis, the pathogen’s immune evasion strategies, and the development of targeted therapeutic interventions targeting these underlying mechanisms.

In this study, we aimed to investigate the interaction between *Tp* and microglia, specifically examining whether autophagy plays a role in processing pathogenic *Tp* by microglia and exploring the underlying mechanism.

## Results

### 1. *Tp* infection decreases microglial numbers in Tg (mpeg1: EGFP) transgenic zebrafish embryos

**Fig 1:**
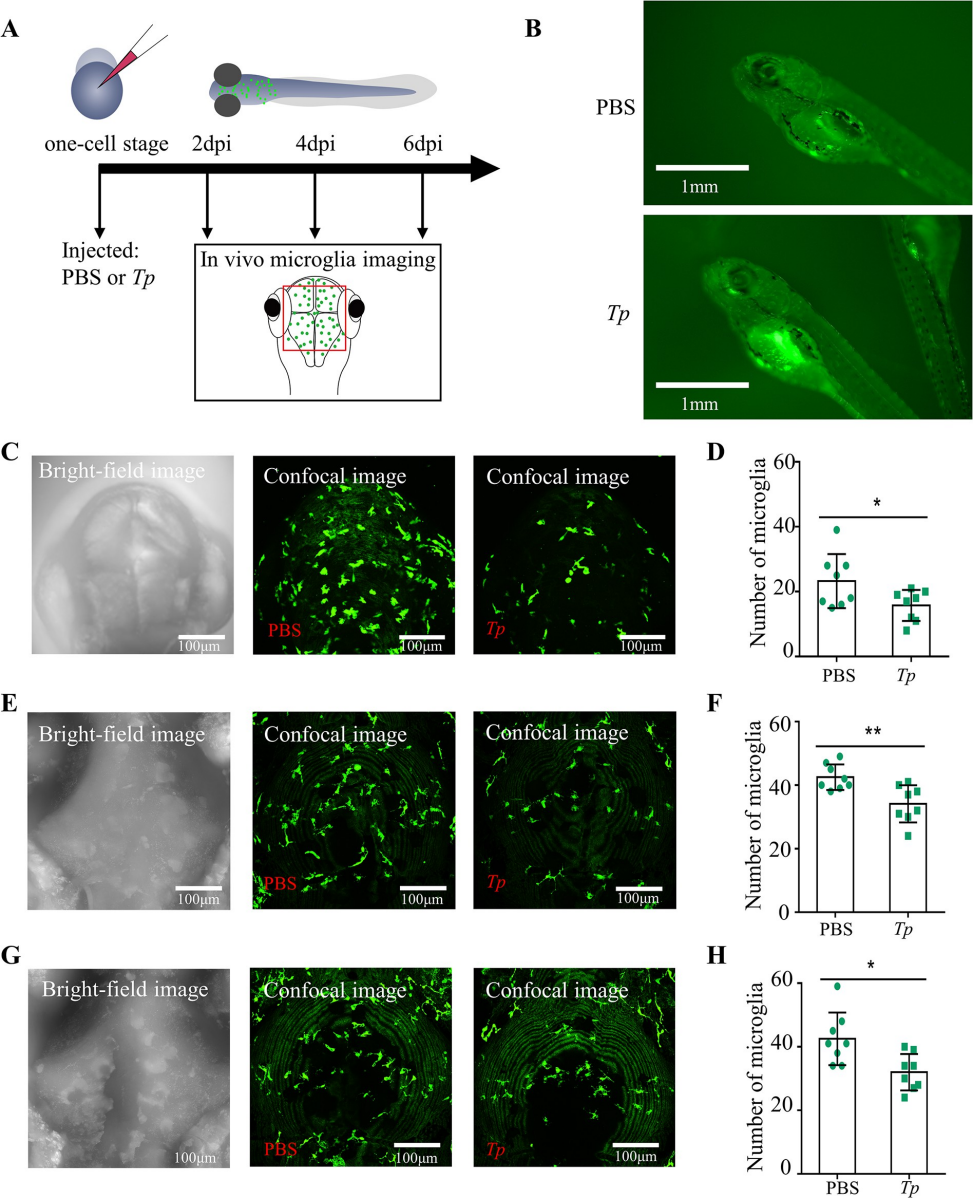
*Treponema pallidum* (*Tp*) infection decreases the numbers of microglia in Tg(mpeg1: EGFP) zebrafish embryos. The number of microglia in *Tp*-treated Tg(mpeg1: EGFP) transgenic zebrafish embryos (*Tp* group) and untreated Tg(mpeg1: EGFP) transgenic zebrafish embryos (Control group) was counted at 2, 4, and 6 days postinfection (dpi). (A) Scheme of the experimental set up. Live one-cell stage Tg (mpeg1: EGFP) transgenic zebrafish embryos were exposed to either 0.1 μL PBS (Control group) or 0.1 μL of 10^9^/mL *Tp* for 2, 4, and 6 days and then imaged with a confocal microscope. The region of interest is framed in red. (B) Tg (mpeg1: EGFP) transgenic zebrafish embryos injected with *Tp* revealed no developmental defect at 4dpi. Scale bar  =  1 mm. (C) Bright-field image and Confocal images of Tg (mpeg1: EGFP) transgenic zebrafish embryos at 2 dpi. Scale bar = 100 μm. (D) The number of microglia of Tg (mpeg1: EGFP) transgenic zebrafish embryos at 2 dpi. (E) Bright-field image and Confocal images of Tg (mpeg1: EGFP) transgenic zebrafish embryos at 4 dpi. Scale bar = 100 μm. (F) The number of microglia of Tg (mpeg1: EGFP) transgenic zebrafish embryos at 4 dpi. (G) Bright-field image and Confocal images of Tg (mpeg1: EGFP) transgenic zebrafish embryos at 6 dpi. Scale bar = 100 μm. (H) The number of microglia of Tg (mpeg1: EGFP) transgenic zebrafish embryos at 6 dpi. The data are shown as the mean ± SD, *P <0.05, n = 8.

In this study, the effects of *Tp* on microglia in vivo was evaluated by injecting Tg (mpeg1: EGFP) transgenic zebrafish embryos with 0.1 μL of 10^9^/mL *Tp*, and embryonic growth was monitored (Fig 1A). No developmental abnormalities were observed in *Tp*-treated embryos (Fig 1B).

At 2, 4, and 6 days post-infection (dpi), a notable decrease in the quantity of microglia was observed in the *Tp*-treated group of Tg (mpeg1: EGFP) zebrafish embryos (n = 8) when compared to the group of untreated embryos (n = 8) (Fig 1C–1H, *P* < 0.05). These findings strongly indicate that *Tp* exhibits substantial toxic effects on microglia in an in vivo setting.

### 2. *Tp* induces microglial apoptosis and activation in vitro

**Fig 2:**
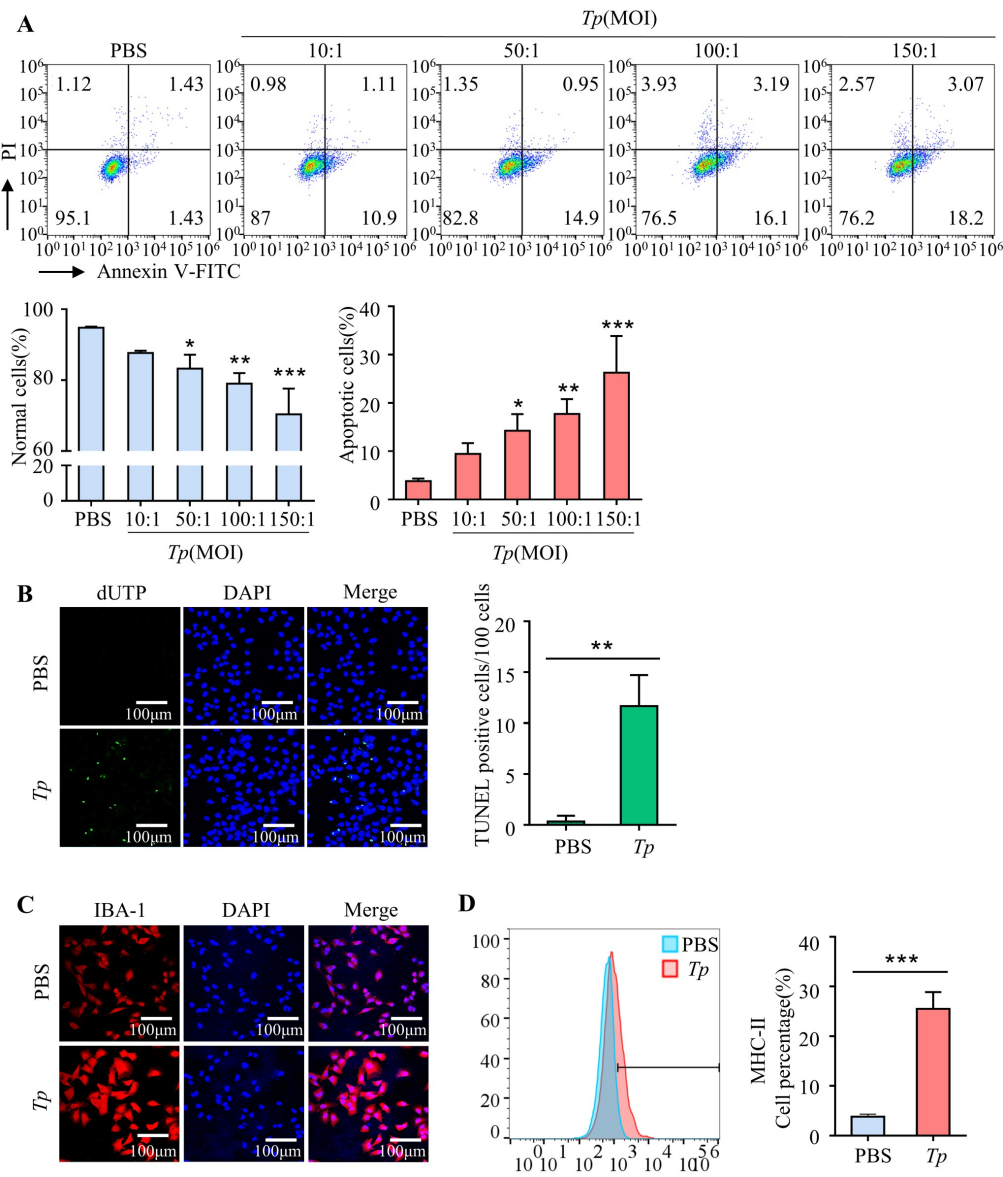
*Tp* induces microglial apoptosis and activation in vitro. The percentage of apoptotic cells was significantly increased in HMC3 cells treated with *Tp* compared to untreated HMC3 cells, and the percentage of normal cells was significantly decreased after *Tp* infection in a dose-dependent manner (MOI of 10:1, 50:1, 100:1 or 150:1). (A) HMC3 cells were treated with *Tp* (MOI of 10:1, 50:1, 100:1, or 150:1) for 24 h. The percentages of apoptotic cells and living cells were analysed by flow cytometry. (B) Apoptosis assay of HMC3 cells after *Tp* treatment at an MOI of 50:1 for 24 h by TUNEL staining. Scale bar  =  100 μm. (C) HMC3 cells were treated with *Tp* (MOI of 50:1) for 24 h and immunostained for IBA. Scale bar  =  100 μm. (D) HMC3 cells were treated with *Tp* (MOI of 50:1) for 24 h, and flow cytometry was used to analyse MHC-II expression. The data are shown as the mean ± SD. *P < 0.05, **P < 0.01, ***P < 0.001.

Microglia can undergo programmed cell death (apoptosis) in response to various stimuli, this can lead to a decrease in the number of microglia in the brain. Hence, the effect of *Tp* on apoptosis was investigated by culturing HMC3 cells with *Tp* (MOI of 10:1, 50:1, 100:1, or 150:1) for 24 h. Flow cytometry analysis revealed that the proportion of apoptotic cells increased in a dose-dependent manner, while the number of normal cells decreased (*P* < 0.05, Fig 2A), a finding consistent with TUNEL staining results (*P* < 0.01, Fig 2B). Taken together, these findings suggest that *Tp* induces microglial apoptosis.

Additionally, to evaluate the activation of microglia by *Tp*, HMC3 cells were exposed to *Tp* (MOI of 50:1), with IBA-1 and MHC-II serving as microglial activation markers. The results showed that *Tp* treatment significantly increased IBA-1 and MHC-II expression in HMC3 cells compared to untreated cells (*P* < 0.001, Fig 2C and 2D), indicating *Tp*-induced microglial activation.

### 3. RNA sequencing revealed that *Tp* induces activation, inhibits autophagy, and promotes apoptosis in HMC3 cells

To elucidate the impact of *Tp* on HMC3 cells, we conducted three independent replicates of RNA sequencing, which enabled the analysis of gene transcription patterns in *Tp*-treated HMC3 cells.

**Fig 3:**
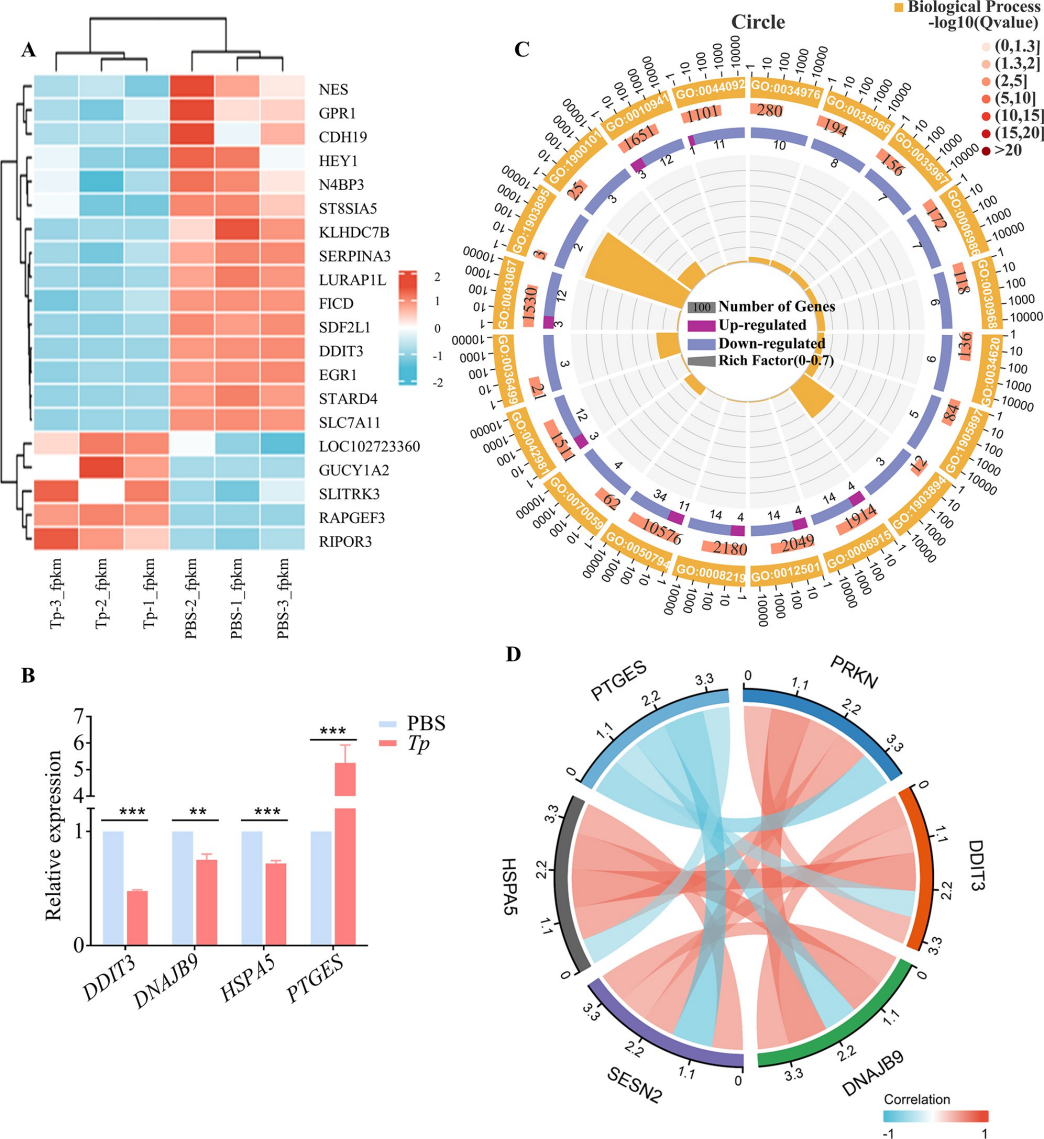
RNA sequencing revealed that *Tp* induces microglial activation and pathogenicity. The gene transcription of HMC3 cells treated with *Tp* and untreated HMC3 cells was analysed by using RNA sequencing. (A) Heatmap showing hierarchical clustering of differentially expressed mRNAs. Red indicates relatively high expression, and blue represents relatively low expression. (B) Four genes were randomly selected to verify the results of transcriptome analysis by real-time qPCR. **P < 0.01, ***P < 0.001, n = 3. (C) GO enrichment analysis. (D) Correlation analysis of apoptotic gene and autophagy signalling pathway gene expression data from RNA-Seq.

Based on the RNA expression levels in the microarray analysis, a hierarchical clustering analysis was performed on the top 20 up/downregulated mRNAs that were associated with apoptosis and autophagy. The dendrogram in Fig 3A demonstrates the different mRNA expression profiles of HMC3 cells acquired from *Tp*-treated HMC3 cells and untreated HMC3 cells. In this analysis, *Tp*-treated HMC3 cells were characterized by the expression of apoptotic genes, such as *PTGES*, with markedly decreased expression levels of autophagy-related genes, such as *DDIT3*, *HSPA5*, and *SESN2*; these results were consistent with the microglial activation phenotype. Four genes were randomly selected for real-time quantitative polymerase chain reaction (qPCR) verification, and the results were consistent with the transcriptome analysis results, which further confirmed the remarkable upregulated of apoptosis-related genes and downregulation of autophagy-related genes (Fig 3B).

Gene Ontology (GO) enrichment analysis showed that the upregulated genes in the *Tp* treatment group were enriched for those that function in apoptotic process, regulation of apoptotic process, regulation of cell death, cell death, etc., while the enrichment of downregulated genes was associated with response to endoplasmic reticulum stress, intrinsic apoptotic signalling pathway in response to endoplasmic reticulum stress, etc. (Fig 3C).

Correlation analysis showed that *Tp*-regulated apoptotic gene expression in HMC3 cells was negatively associated with autophagy signalling pathway-related gene expression (Fig 3D). This result indicated that *Tp* promotes microglial activation, inhibits autophagy, and induces cell apoptosis.

### 4. *Tp* blocks the autophagic flux in HMC3 cells

**Fig 4:**
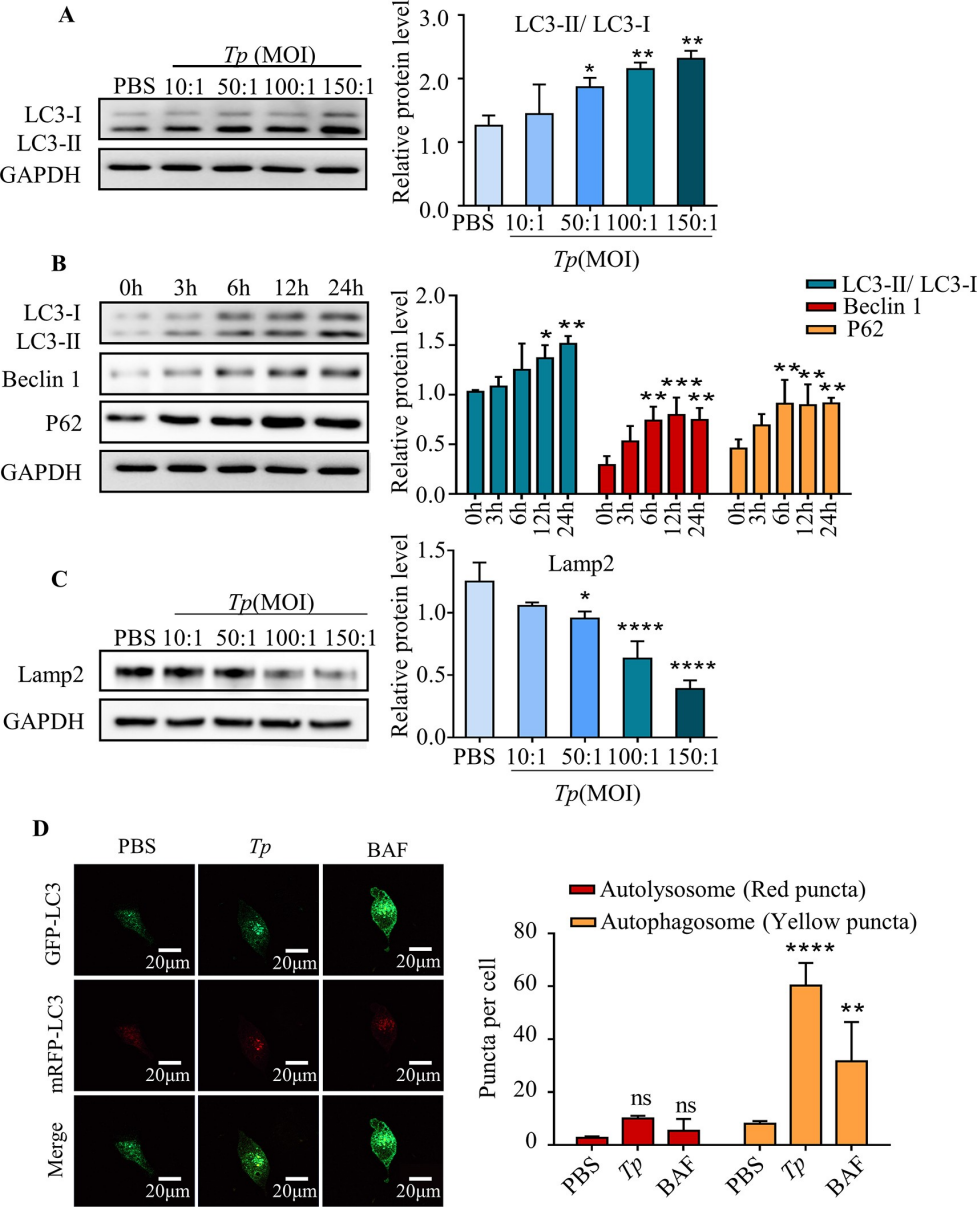
*Tp* blocks the autophagic flux. A tandem fluorescent-labelled plasmid pmCherry-EGFP-LC3B construct was transfected into HMC3 cells to quantify the numbers of different LC3 puncta and to assess the impact of *Tp* on the autophagic flux in HMC3 cells. In the absence of autophagy, the cells emitted diffuse yellow fluorescence. GFP (green fluorescence) is sensitive to the acidic environment of the lysosome and mCherry (red fluorescent) exhibits significant stability. After the initiation of autophagy, yellow puncta (RFP+ GFP+) indicated early autophagosomes, whereas red puncta (RFP+ GFP-) indicated late autophagosomes, showing that LC3 had been delivered to the lysosomes and that autophagosomes and lysosomes had fused to form autolysosomes. (A) The expression of LC3-I and LC3-II after HMC3 cells were treated with different concentrations of *Tp* for 24 h was measured by Western blotting. (B) Expression of the autophagy-related proteins Beclin1, LC3-I, LC3II and P62 after HMC3 cells were treated with *Tp* (MOI of 50:1) for different times was measured by Western blotting. (C) Expression of Lamp2 after HMC3 cells were treated with different concentrations of *Tp* for 24 h was measured by Western blotting. (D) HMC3 cells were transfected with the pmCherry-EGFP-LC3B plasmid, cocultured with or without *Tp* (MOI of 50:1) for 24 h, and analysed by confocal fluorescence microscopy. HMC3 cells were treated with BAF (bafilomycin, 80 nM) for 24 h as a positive control. Scale bar: 20 μm. The data are presented as the mean ± SD of three independent experiments. *P < 0.05, **P < 0.01, ***P < 0.001.

To investigate the effect of *Tp* on autophagy in microglial, HMC3 cells were cultured with *Tp* (MOI of 10:1, 50:1, 100:1, or 150:1) for 24 h. Western blotting results showed that the conversion of LC3-I to LC3-II dramatically increased in a dose-dependent manner (*P* < 0.01), indicating that *Tp* could induce autophagy in HMC3 cells (Fig 4A).

In addition to the initiation of autophagy, the accumulation of autophagosomes may also be caused by inhibition of the autophagy flux and defective degradation ability [12]. Hence, the levels of autophagy-related proteins were examined. Western blotting results showed that the *Tp*-induced accumulation of P62 increased with the LC3-II/ LC3-I ratio and Beclin1 protein level (*P* < 0.01, Fig 4B), suggesting either impaired lysosome/autolysosome function or decreased lysosomal numbers.

In addition, the level of lysosome-associated membrane glycoprotein 2 (Lamp2) was evaluated and results showed that the number of lysosomes dramatically decreased in a dose-dependent manner in HMC3 cells (*P* < 0.01, Fig 4C), suggesting that *Tp* is an inhibitor of lysosomal biogenesis.

A tandem fluorescent-labelled plasmid pmCherry-EGFP-LC3B construct was transfected into HMC3 cells to assess the effect of *Tp* on the autophagic flux in HMC3 cells. As shown in Fig 4D, the quantity of LC3B puncta was significantly increased in *Tp*-treated cells (*P* < 0.001), and most of the spots emitted RFP+ GFP+ signals (autophagosomes, yellow puncta) rather than RFP+ GFP- signals (autolysosomes, red puncta), indicating the increased accumulation of autophagosomes and the decreased formation of autolysosomes. Bafilomycin A1 (BAF; autophagosome-lysosome fusion inhibitor) as the positive control.

These results suggest that *Tp* infection resulted in an insufficient number of lysosomes for fusion with autophagosomes, which probably contributes to the *Tp-*induced inhibition of the autophagic flux in HMC3 cells.

### 5. *Tp* blocks the autophagic flux in HMC3 cells by inhibiting transcription factor EB (TFEB) nuclear translocation via mTORC1 signaling activation

To explore the mechanism by which *Tp* inhibits lysosomal biogenesis in HMC3 cells, the mTORC1/TFEB axis was analysed.

**Fig 5:**
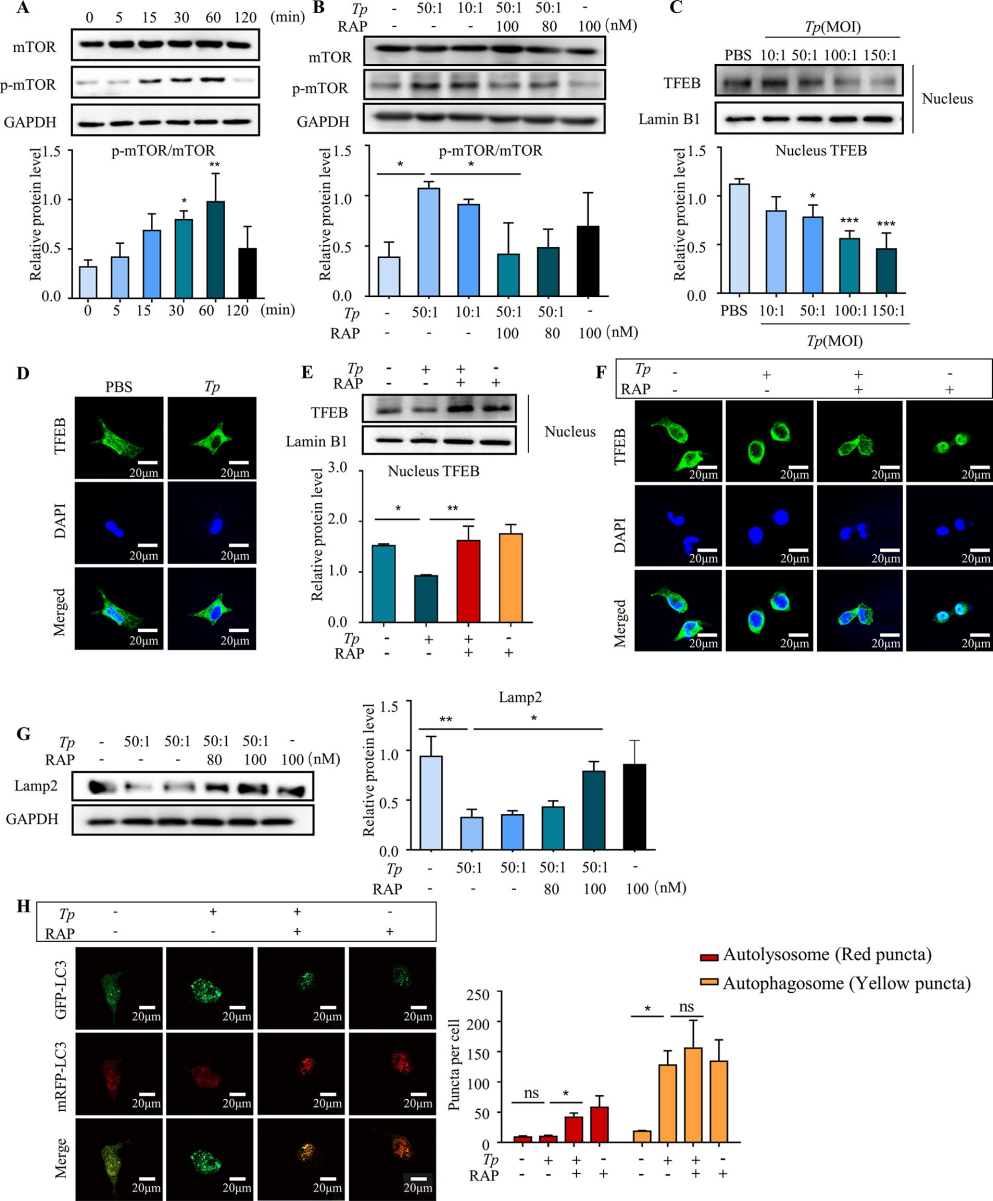
*Tp* blocks the autophagic flux in HMC3 cells by inhibiting TFEB nuclear translocation and reducing lysosomal biosynthesis via activation of the mTORC1 signalling pathway. To assess mTOR activation in *Tp*-treated HMC3 cells, HMC3 cells were cultured with *Tp* (MOI of 50:1) for different times, and Western blotting was utilized to analyse mTOR signalling pathway components. To investigate the effect of mTORC1 signalling on lysosome biosynthesis and TFEB transcription activity, Western blotting and immunostaining were utilized to measure the expression of lysosome-associated membrane glycoprotein 2 (Lamp2) and nuclear levels of TFEB. To investigate the effect of mTORC1 signalling on the autophagic flux, a tandem fluorescent-labelled plasmid pmCherry-EGFP-LC3B construct was transfected into HMC3 cells. Then, HMC3 cells were pretreated with RAP (rapamycin, mTORC1 inhibitor, 100 nM) for 2 h and cocultured with *Tp* (MOI of 50:1) for 24 h. Finally, the results were observed under a confocal microscope. (A) Total mTOR protein levels and phosphorylated mTOR protein levels after HMC3 cells were treated with *Tp* (MOI of 50:1) for different times were measured by Western blotting. (B) HMC3 cells were pretreated with the mTORC1 signalling inhibitor RAP (rapamycin, 100 nM) for 2 h and then cocultured with *Tp* (MOI of 50:1) for 1 h. The effects of RAP on mTOR signalling were measured by Western blotting. (C) The nuclear levels of TFEB after HMC3 cells were treated with different concentrations of *Tp* for 24 h was measured by Western blotting. (D) HMC3 cells were treated with *Tp* (MOI of 50:1) for 24 h and immunostained for TFEB. Scale bar  =  100 μm. (E) HMC3 cells were pretreated with the mTORC1 signalling inhibitor RAP (rapamycin, 100 nM) for 2 h and then cocultured with *Tp* (MOI of 50:1) for 24 h. The effects of RAP on the nuclear levels of TFEB were measured by Western blotting. (F) HMC3 cells were pretreated with the mTORC1 signalling inhibitor RAP (rapamycin, 100 nM) for 2 h, cocultured with *Tp* (MOI of 50:1) for 24 h, and immunostained for TFEB. Scale bar  =  20 μm. (G) HMC3 cells were pretreated with the mTORC1 signalling inhibitor RAP (rapamycin, 100 nM) for 2 h and then cocultured with *Tp* (MOI of 50:1) for 24 h. The effects of RAP on Lamp2 expression were measured by Western blotting. (H) HMC3 cells were transfected with the pmCherry-EGFP-LC3B plasmid, pretreated with the mTORC1 signalling inhibitor RAP (rapamycin, 100 nM) for 2 h and cocultured with *Tp* (MOI of 50:1) for 24 h. The effects of RAP on the changes in the autophagic flux in HMC3 cells were analysed by confocal fluorescence microscopy. Scale bar: 20 μm. The data are presented as the mean ± SD of three independent experiments. *P < 0.05, **P < 0.01, ***P < 0.001.

First, HMC3 cells were cultured with *Tp* (MOI of 50:1) for different times (0 min, 5 min, 15 min, 30 min, 60 min, or 120 min) to assess mTOR activation in *Tp*-treated HMC3 cells. Compared with HMC3 cells treated with *Tp* for 0 h, the ratio of p-mTOR (Ser2448)/mTOR was significantly increased after 15 min (*P* < 0.05) and peaked at 60 min (*P* < 0.001, Fig 5A). This indicated that *Tp* markedly increased mTORC1 activity, as shown by the increased levels of mTOR phosphorylated at Ser2448, which is a well-known substrate protein that is phosphorylated by mTORC1.

In addition, western blotting assay and immunostaining assay for TFEB revealed that *Tp* markedly decreased the nuclear levels of TFEB in HMC3 cells (*P* < 0.05, Fig 5C and 5D). Rapamycin (RAP) is a potent mTORC1 inhibitor that can significantly inhibit the *Tp-*induced protein expression of p-mTOR (Ser2448) (*P* < 0.05, Fig 5B). All these *Tp*-induced changes in TFEB localization were reversed by RAP treatment (100 nM) (*P* < 0.01, Fig 5E and 5F). Furthermore, the *Tp* (MOI of 50:1)*-*induced decrease in the Lamp2 protein levels were partially reversed by RAP treatment (100 nM) (*P* < 0.05, Fig 5G). These results demonstrated that the *Tp-*mediated inhibition of TFEB nuclear translocation is mTORC1 dependent, and the decreased Lamp2 levels in the lysosomal fractions of *Tp*-treated HMC3 cells could be due to decreased lysosomal biogenesis as a result of TFEB inactivation.

To further verify that the mTOR/TFEB axis contributed to the *Tp*-induced inhibition of the autophagic flux, a tandem fluorescent-labelled plasmid pmCherry-EGFP-LC3B construct was transfected into HMC3 cells. The results showed that the numbers of red puncta (autolysosomes) in HMC3 cells cotreated with *Tp* and RAP (100 nM) were markedly increased compared with those in HMC3 cells treated with *Tp* alone (P < 0.05, Fig 5H); these results indicated that the inhibition of mTORC1 can promote lysosomal biogenesis to reverse the *Tp-*induced inhibition of the autophagic flux.

### 6. *Tp* promoted microglia apoptosis and prevented itself from clearing by HMC3 cells via causing autophagosome accumulation

**Fig 6:**
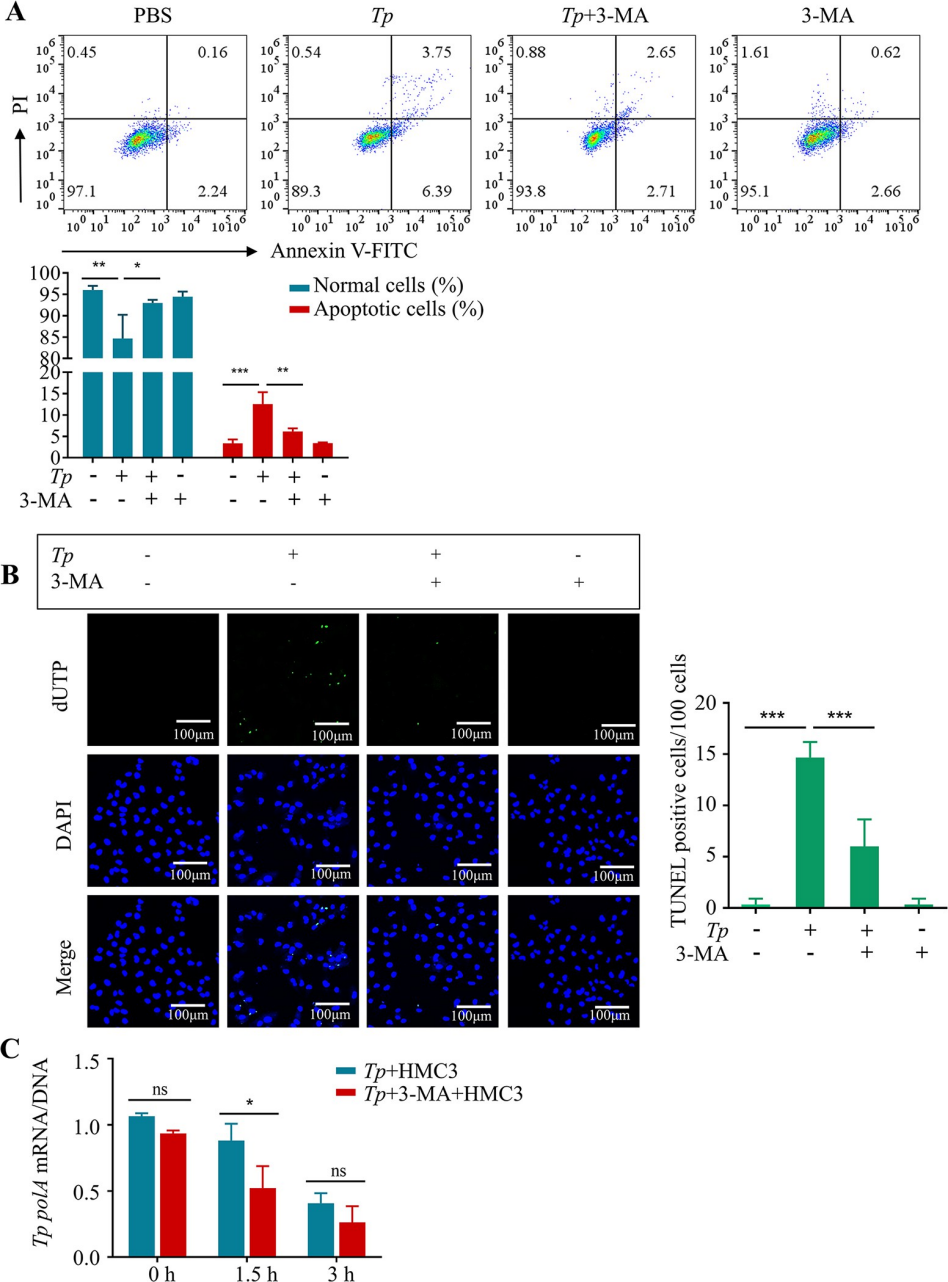
The accumulation of autophagosomes is associated with the mechanism by which *Tp* induces apoptosis and inhibits its own clearance in HMC3 cells. (A) HMC3 cells were treated with *Tp* (MOI of 50:1) with or without 3-MA (3-methyladenine, autophagosome formation inhibitor, 2 mM) for 24 h. The percentages of apoptotic cells and live cells were analysed by flow cytometry. (B) HMC3 cells were treated with *Tp* (MOI of 50:1) with or without 3-MA (3-methyladenine, autophagosome formation inhibitor, 2 mM) for 24 h. Apoptosis was analysed by TUNEL staining. Scale bar  =  100 μm. (C) HMC3 cells were treated with *Tp* (MOI of 10:1) with or without 3-MA (2 mM) for different times. The *polA* mRNA/DNA ratio was determined. The data are presented as the mean ± SD of three independent experiments. *P < 0.05, **P < 0.01, ***P < 0.001.

To determine whether the accumulation of autophagosomes is a significant factor in *Tp-*induced apoptosis in HMC3 cells, the inhibitor 3-methyladenine (3-MA) was used to inhibit autophagosome formation. Flow cytometry results showed that combined treatment with *Tp* and 3-MA significantly decreased the percentage of apoptotic cells compared with *Tp* alone (*P* < 0.01, Fig 6A), which was consistent with the TUNEL staining results (*P* < 0.01, Fig 6B).

In addition, it is well known that microglia clear and kill foreign pathogens. To evaluate whether the accumulation of autophagosomes is associated with the ability of microglia to clear *Tp*, HMC3 cells and *Tp* (MOI of 10:1) were cocultured. Real-time qPCR was used to analyse the expression levels of *Tp polA* DNA and *Tp polA* mRNA inside and outside of the cells at different time points (0 h, 1.5 h, and 3 h), and the *Tp polA* mRNA/DNA ratio was calculated to analyse the activity of *Tp*. The results showed that the activity of *Tp* gradually decreased with the extension of coculture time. After coculture for 3 h, the group treated with the combination of *Tp* and 3-MA exhibited significantly reduced *Tp* activity compared with the group treated with *Tp* alone, indicating that inhibition of autophagosome accumulation can promote the clearance of *Tp* by microglia (*P* < 0.05, Fig 6C).

Our results indicated that *Tp* induces apoptosis and inhibits its own clearance in HMC3 cells by blocking the autophagic flux and causing the accumulation of autophagosomes.

## Discussion

The well-recognized capacity of *Tp* to evade the immune system has led to its designation as “the stealth pathogen” [13]. However, the role of *Tp* in microglia and the underlying mechanisms remain largely unknown. In this study, we conducted an investigation into the dynamic interaction between *Tp* and microglia. Our findings demonstrate that *Tp* exerts a significant impact on microglial populations, leading to a notable decrease in cell numbers. Furthermore, we observed a pronounced promotion of microglial apoptosis induced by *Tp*. Additionally, our study revealed a remarkable phenomenon wherein *Tp* employs a mechanism to impede its clearance by microglia. These observations collectively shed light on the intricate interplay between *Tp* and microglial cells, providing valuable insights into the pathogenesis of *Tp* infection. Our findings provide novel evidence for the immune evasion of *Tp* in microglia and shed light on the mechanism underlying this process.

Autophagy dysregulation and neuroinflammation are closely related to the development of neurodegeneration. In our study, the inhibitory effect of *Tp* on HMC3 cell autophagy relates genes was observed by RNA sequencing. In addition, the *Tp*-induced accumulation of P62 was provided further support for autophagy impairment. More importantly, a decrease in the Lamp2 protein levels demonstrated that *Tp* inhibited lysosomal biogenesis, and transient transfection with the pmCherry-EGFP-LC3B plasmid confirmed that *Tp* inhibited the fusion between autophagosomes and lysosomes. These data indicated that *Tp* blocks the autophagic flux in HMC3 cells by inhibiting lysosomal biogenesis. It has been reported that autophagy may regulate LPS-stimulated inflammation in microglia and its associated neurotoxicity [14,15]. Autophagy dysregulation may be a hallmark of *Tp* neuropathogenesis.

TFEB is a master regulator of lysosome biogenesis and autophagy-related gene transcription and mainly regulated by mTORC1 [16,17]. We found that *Tp* increased mTORC1 activation in HMC3 cells. Consistent with the known negative regulatory effect of mTORC1 on TFEB, *Tp* decreased TFEB nuclear translocation. RAP, which is a specific mTORC1 inhibitor, reversed the *Tp*-induced decrease in the nuclear levels of TFEB, further suggesting a critical role of mTORC1 in regulating the *Tp*-mediated inactivation of TFEB. Increasing evidence demonstrates that decreased TFEB activity increases neurotoxicity and promotes neurodegeneration. In some neurodegenerative disorders, including polyglutamine disease [18] and Parkinson’s disease [19], pathogenesis is exacerbated by decreasing TFEB function, which impairs autophagy. To further determine whether TFEB disorder is related to *Tp*-induced autophagy impairment, the effect of the mTORC1/TFEB axis on *Tp*-treated HMC3 cells was investigated. We found that the inhibition of mTORC1 reversed the *Tp*-induced decrease in lysosome biosynthesis and impaired autophagy flux, which indicated that the *Tp-*mediated inactivation of TFEB resulted in insufficient numbers of lysosomes for fusion with autophagosomes, leading to autophagy arrest and autophagosome accumulation.

In previous studies, inhibition of the autophagic flux and accumulation of autophagosomes were shown to lead to apoptosis [20–23], and such findings were consistent with those of the present study. In the present study, RNA sequencing revealed that *Tp*-regulated apoptotic gene expression in HMC3 microglia was negatively associated with autophagy signalling pathway-related gene expression. In vitro cell experiments showed that *Tp*-induced apoptosis was significantly reversed by treatment with the autophagosome formation inhibitor 3MA. Based on those findings, *Tp*-induced autophagosome accumulation was shown to be the cause of the proapoptotic effects in microglia. Notably, we also found that *Tp* decreased the number of microglial cells in Tg (mpeg1: EGFP) transgenic zebrafish embryos, which may be mediated by *Tp*-induced microglial cell apoptosis.

Many studies have reported that macrophages are closely related to the clearance of *Tp* [5,6,24,25]. We further investigated whether blocking the autophagic flux would affect the killing and elimination of *Tp* by microglia. Bacterial mRNA generally has an extremely short half-life and is more vulnerable to destruction than rRNA or genomic DNA [26–28]. Therefore, a positive mRNA signal indicates the presence of a recently viable organism. Craig Tipple *et al*. showed that the half-life of *Tp* DNA was significantly longer than that of RNA [29]. Thus, real-time qPCR was used to analyse *Tp polA* DNA expression levels to evaluate total numbers of *Tp*, *Tp polA* mRNA expression levels were used to evaluate the activity of *Tp*, and the mRNA/DNA ratio was used to determine the survival rate of *Tp* to analyse the clearance of *Tp* by HMC3 cells. The results indicated that the survival rate of *Tp* decreased gradually with the extension of coculture time, and 3-MA significantly reduced the survival rate of *Tp*, indicating that 3-MA could promote the clearance of *Tp* by HMC3 cells. Studies have shown that microglia can clear pathogenic bacteria via autophagy [30]. However, in this study, the autophagy inhibitor 3-MA promoted the clearance of *Tp* by microglia. Previous studies have shown that increased apoptosis is responsible for the incomplete clearance of *Tp* from lesions and chronic infections [31], and we hypothesize that 3-MA may promote the clearance of *Tp* by inhibiting apoptosis.

Despite the significant findings and initial insights obtained from this study regarding the interplay between *Tp* and microglial cells, it is crucial to acknowledge the presence of certain limitations. Firstly, the study employed in vitro cell experiments and in vivo animal models to simulate *Tp* infection, yet it is important to recognize that these models do not fully recapitulate the intricate physiological and pathological processes occurring within the human body. Secondly, the observed decrease in the number of microglial cells may be influenced by factors beyond apoptosis, such as the inhibition of cell proliferation or the migration of other immune cells. Consequently, further investigations are warranted to explore additional potential factors contributing to the reduction of microglial cells induced by *Tp*. This will foster a more comprehensive understanding of the complex interaction between *Tp* and microglial cells, while aiding in the identification of prospective therapeutic targets for modulating microglial function and mitigating the detrimental consequences of *Tp* infection on the central nervous system.

**Fig 7:**
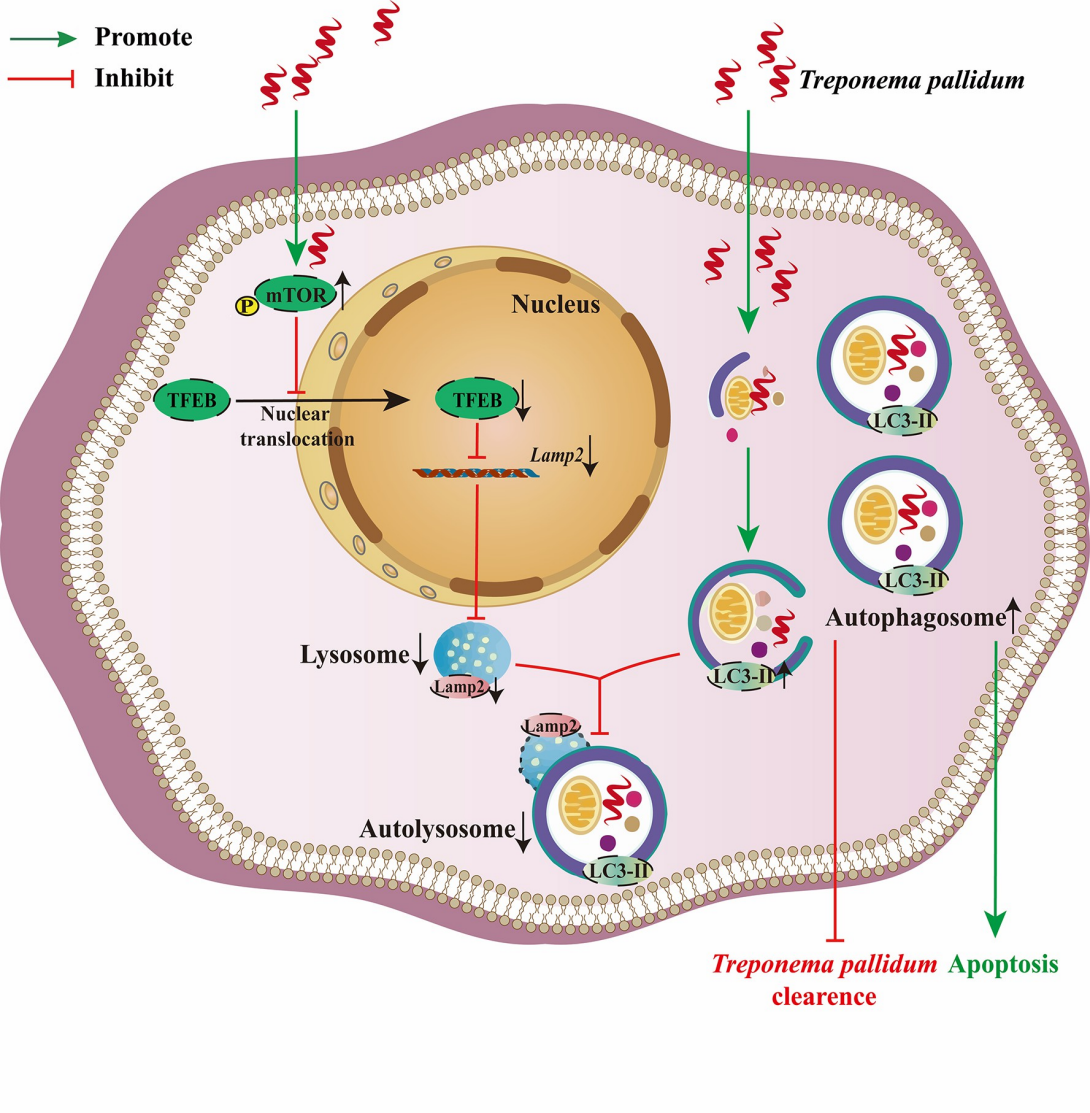
*Tp* promoted microglia apoptosis and prevented itself from clearing by human microglia via blocking autophagic flux. In this study, we demonstrated that *Tp* activated mTORC1 signalling to inhibit the nuclear translocation of transcription factor EB (TFEB), decreasing lysosomal biogenesis and causing autophagy arrest and autophagosome accumulation. Autophagosome accumulation was demonstrated to be a key mechanism by which *Tp* promoted microglia apoptosis and prevented itself from clearing by human microglia via blocking autophagic flux.

In conclusion, our present in vitro and in vivo study results provided robust evidence that supported the toxic effect of *Tp* on microglia. *Tp* blocks the autophagy flux by inhibiting TFEB-mediated lysosomal biosynthesis in human microglia, and autophagosome accumulation was demonstrated to be a key mechanism by which *Tp* induces apoptosis and inhibits its clearance in human microglia. Our findings provide insights into the possible mechanism underlying the effect of *Tp* on microglial autophagy and may provide novel evidence for understanding the pathogenesis of neurosyphilis (Fig 7).

## Materials and Methods

### 1. Ethics statement

All the experiments that involved animals were approved by the Laboratory Animal Management and Ethics Committee of Xiamen University.

### 2. Animal studies

Zebrafish were obtained from Hunter Biotech’s laboratory (Hangzhou, China). The transgenic lines Tg(mpeg1: EGFP) were described previously [32]. All the experiments that involved zebrafish were approved by the Laboratory Animal Management and Ethics Committee of Xiamen University.

The Tg (mpeg1: EGFP) transgenic zebrafish embryos in the control group were injected with 0.1 μL PBS, and the Tg (mpeg1: EGFP) transgenic zebrafish embryos in the experimental group were injected with 0.1 μL *Tp* (10^9^/mL). At 22 hours postinfection (hpi), the zebrafish embryos were treated with 0.003% 1-phenyl-2-thiourea (PTU) to inhibit pigmentation.

At 2, 4, and 6 days post-infection (dpi), the zebrafish embryos were mounted in 1.5% low-melting-point agarose in distilled water. Images were captured using a confocal microscope (AX, Nikon, Japan).

### 3. Cell culture and treatment

The human microglial clone 3 (HMC3) cell line was obtained from the American Type Culture Collection (ATCC, USA). The cells underwent cultivation in a specialized medium (Procell Life Science & Technology Co., Ltd., Wuhan, China) supplemented with 10% fetal bovine serum and 1% penicillin/streptomycin. Subsequently, the cells were incubated under specific conditions in a humidified incubator at a temperature of 37°C, while maintaining a 5% CO2 atmosphere.

The HMC3 cells were subjected to a 24-hour treatment with BAF (80 nM). Furthermore, prior to coculturing with *Tp* (MOI of 50:1) for 24 hours, the cells underwent a 2-hour pretreatment with either RAP (100 nM) or 3-MA (2 mM). The BAF (A8510) compound was procured from Solarbio, Beijing, China, while RAP (HY-10219) and 3-MA (HY-19312) were obtained from Med Chem Express, USA.

### 4. Preparation of virulent *Tp*

The *Tp* Nichols strain used in this study was generously provided by Lorenzo Giacani, Ph.D. (University of Washington, Seattle, WA, USA) and maintained for virulence through intratesticular propagation in adult male New Zealand white rabbits, following previously described methods [33,34].

After infection of the rabbits with *Tp*, euthanasia was performed, and *Tp* was extracted from the enlarged infected testicles using a warm saline solution (0.9%). Aseptic excision of the testicles was conducted, and longitudinal incisions were made on each testicle. The testicular tissue was then immersed in a warm saline solution (0.9%) and subjected to vigorous shaking on a shaker. The gross testicular debris was sedimented through low-speed centrifugation (1000⊆g, 15 minutes), and the resulting supernatant underwent high-speed centrifugation (16000⊆g, 30 minutes) to obtain the *Tp* pellet. Subsequently, the *Tp* pellet was resuspended in PBS and thoroughly mixed. To further eliminate any remaining impurities, the resuspended pellet underwent an additional round of low-speed centrifugation (1000⊆g, 15 minutes). The resulting supernatant was subjected to high-speed centrifugation (16000⊆g, 30 minutes) to obtain the final *Tp* pellet. The *Tp* pellet was resuspended in 1 mL of PBS, thoroughly mixed, and examined under a dark-field microscope at 40⊆ magnification to visualize the elongated spiral-shaped *Tp* organisms. Enumeration was performed, and the organisms were prepared for subsequent usage.

In a similar manner, testicles from uninfected rabbits underwent aseptic excision and followed the same extraction process described above for *Tp* purification. In this specific case, the resulting PBS solution did not contain *Tp* but could potentially contain residual rabbit tissue. This PBS solution was utilized as a control to eliminate any potential interference from residual rabbit tissue on the experimental results.

### 5. Immunofluorescence assay and TUNEL assay

HMC3 cells were seeded in 24-well plates with glass-bottom cover slips.

For the immunofluorescence assay, the HMC3 cells were subjected to an overnight incubation with goat monoclonal antibodies to IBA-1 (1:200, Abcam, Britain) and rabbit monoclonal antibodies to TFEB (1:100, Abcam, Britain). Subsequently, the cells were incubated with Alexa Fluor 594-conjugated donkey anti-goat IgG (1:500, Invitrogen, USA) and Alexa Fluor 488-conjugated goat anti-rabbit IgG (1:500, Abcam, Britain) for 1 hour. To visualize the nuclei, DAPI staining (Beyotime, Shanghai, China) was performed for a duration of 10 minutes.

For the TUNEL assay, the HMC3 cells were subjected to an incubation period of 1 hour in the dark with a mixture consisting of 45 μL of fluorescent labeling solution containing dUTP-biotin and 5 μL of TdT enzyme (Beyotime, Shanghai, China). The objective of this procedure was to enable the visualization and detection of DNA fragmentation in apoptotic cells by means of a combination of fluorescent labeling and probes. Additionally, the cell nuclei were stained with DAPI (Beyotime, Shanghai, China) for a duration of 10 minutes.

Images were visualized using confocal laser scanning microscopy (LSM780, Zeiss, Germany).

### 6. Flow cytometry assay

HMC3 cells were seeded in 6-well plates at a density of 1 × 10^5^ cells per well.

For the analysis of cell surface expression of MHC-II, a staining procedure was performed using 10 μL of anti-MHC-II antibodies (mouse anti-human HLADR-FITC) for a duration of 30 minutes under dark conditions. A gating strategy was implemented to exclude debris and doublets based on the forward scatter (FSC) and side scatter (SSC) properties of the cell population. FITC was selected as the excitation light, and FITC-labeled mouse IgG was utilized as the isotype control to evaluate non-specific binding and establish the gating boundaries. The assessment of MHC-II expression was conducted exclusively within the viable cell population.

To analyse apoptosis, cells were resuspended in binding buffer and stained with 5 μL Annexin V-FITC plus 10 μL propidium iodide (Beyotime, Shanghai, China) at 4°C for 10 minutes in the dark.

The percentage of MHC-II-positive cells and apoptotic cells was analysed by using flow cytometry (Beckman Coulter, USA) within 1 h.

### 7. Western blotting (WB) assay

HMC3 cells were seeded in 6-well plates at a density of 1 × 10^5^ cells per well. After treatment, western blotting assay is performed as described previously [35].

### 8. pmCherry-GFP-LC3B plasmid transfection and colocalization assay

The HMC3 cells were plated in 24-well plates with glass-bottom cover slips and transiently transfected with the pmCherry-EGFP-LC3B plasmid (MiaoLing Biotechnology, Wuhan, China. At the conclusion of a 72-hour incubation period, the cells were subjected to treatment with *Tp* (at a MOI of 50:1) or BAF (at a concentration of 80 nM). After treatment for 24 h, the cells were viewed under an LSM700 confocal laser scanning microscope (Zeiss, Oberkochen, Germany).

After the cells were transfected with the pmCherry-GFP-LC3B plasmid, diffuse yellow fluorescence was observed under a fluorescence microscope (LSM780, Zeiss, Germany) in the absence of autophagy. GFP (green fluorescence) is sensitive to the acidic environment of the lysosome, and mCherry (red fluorescent) exhibits significant stability. After the initiation of autophagy, yellow puncta (RFP+ GFP+) indicated early autophagosomes, whereas red puncta (RFP+ GFP-) indicated late autophagosomes; the presence of red puncta suggested that LC3 had been delivered to lysosomes and that autophagosomes and lysosomes had fused to form autolysosomes. The numbers of red and yellow puncta were counted to help gauge the intensity of the autophagic flux.

### 9. Microarray analysis of RNAs (transcriptome sequencing)

Total RNA was extracted from cultured cells using an RNeasy kit (Tiangen Biotech Co., Ltd., Beijing, China), rRNAs were removed, and mRNAs were enriched. Then reverse transcripted with a high-capacity cDNA reverse transcription kit (Takara, Shiga, Japan) and sequenced using the Illumina HiSeq 2500 platform by Gene Denovo Biotechnology Co (Guangzhou, China). EdgeR and DESeq2 software were used. Analysis of differentially expressed RNA and principal component analysis were performed with R package models (http://www.r-project.org/). Gene Ontology (GO) enrichment analysis provides all GO terms. The fold-change differences in mRNA expression levels were visualized in the heatmap using the OmicShare Platform with sample clustering.

### 10. Real-time fluorescence quantitative PCR

#### 10.1 DNA extraction

Lysis buffer mixed with cell culture supernatant was added to cells and incubated with Proteinase K (QiAmp, Qiagen, Crawley, UK) for 10 min. AFTER extraction with a proprietary extraction method (QiAmp, Qiagen, Crawley, UK), DNA was purified by ethanol precipitation and resuspended in elution buffer (QiAmp, Qiagen, Crawley, UK).

#### 10.2 RNA extraction

Total RNA was extracted from the cultured cells using an RNeasy kit (Tiangen Biotech Co., Ltd., Beijing, China) and then reverse transcribed with a high-capacity cDNA reverse transcription kit (Takara, Shiga, Japan).

#### 10.3 Absolute quantitative RT–PCR assay

Amplification of the *Tp polA* gene was achieved by RT–qPCR assay to quantify *Tp* DNA and RNA levels. Each reaction contained 10×Buffer, Taq Polymerase, MgC12, dNTP mix, nuclease-free water, the cDNA or DNA sample, the primer, and the hydrolysis probe (FAM-TTGCGACGCTGCGTACGTACAGCAACGGT-TAMRA). The cycling conditions and parameters employed in this study were as follows: an initial denaturation step at 95°C for 3 minutes, followed by 45 cycles of denaturation at 95°C for 10 seconds, annealing and extension at 60°C for 30 seconds (signal acquisition), and a final extension step at 37°C for 10 seconds. Quantification was achieved using an ‘in-run’ plasmid standard of a 1:10 dilution series from 10^6^ to 100 copies/μL of the *polA* target sequence. The amplified RT–qPCR products were recovered, perform Sanger sequencing was performed. All the reactions were carried out with a LightCycler 480 II (Roche, Basel, Switzerland). The primers are listed in S1 Table.

#### 10.4 Relative quantitative RT–PCR assay

RT–qPCR was performed using SYBR Green PCR Master Mix (Takara, Shiga, Japan) on a PCR Thermal Cycler MA-6000 (MOLARRAY, Suzhou, China). The expression values were analysed using the 2−ΔΔCt method. The primers are listed in S1 Table.

### 11. Statistical analysis

The data are presented as the mean ± SD of 3 independent experiments. Statistical analysis was conducted using GraphPad Prism 8.0 software (San Diego, California USA, www.graphpad.com), and Student’s t test was used for comparisons between the 2 groups. One-way analysis of variance (ANOVA) was used for comparisons among multiple sets of quantitative data. Differences were considered significant when *P < 0.05, **P < 0.01, or ***P < 0.001.

## Supporting information

S1 Table Primer sequences. (DOCX)
