# Supplementary material for: Treponema pallidum promoted microglia apoptosis and prevented itself from clearing by human microglia via blocking autophagic flux
Source: PLoS Pathog. 2023 Aug 23;19(8):e1011594. doi: 10.1371/journal.ppat.1011594 (PMC10446187; doi:10.1371/journal.ppat.1011594)
Supplement: S1 Table — (DOCX) [file ppat.1011594.s001.docx]

**Table S1. Primer sequences.**

| **ID** | **Sense primer (5ʹ-3ʹ)** | **Antisense primer (5ʹ-3ʹ)** |
| --- | --- | --- |
| *polA* | TACGGTGCAAGTGCTCAGAC | CAGGCACATTGTCGGAGGAA |
| *DDIT3* | GGAAACAGAGTGGTCATTCCC | CTGCTTGAGCCGTTCATTCTC |
| *DNAJB9* | TCTTAGGTGTGCCAAAATCGG | TGTCAGGGTGGTACTTCATGG |
| *HSPA5* | GAAAGAAGGTTACCCATGCAGT | CAGGCCATAAGCAATAGCAGC |
| *PTGES* | TCCTAACCCTTTTGTCGCCTG | CGCTTCCCAGAGGATCTGC |
